# Surgicel as an Unusual Cause of Prolonged Drainage

**DOI:** 10.4103/2006-8808.73626

**Published:** 2010

**Authors:** Vipul Gupta, Abdulnaser Al Said, Sunil Kumar, Ashhad A. Khan

**Affiliations:** *Department of pediatric surgery and urology, IBN SINA Hospital, State of Kuwait E-mail: drvipul75@yahoo.co.in*

Dear Editor,

With introduction of concept like nephron-sparing surgery, partial nephrectomy using surgicel as a haemostatic agent is now being recommended as a procedure of choice for an increasing number of pediatric urology disorders. Although surgicel is considered a safe haemostat, it still has been found to be associated with unusual complications.[1–4] Thus, we herein share our unique experience with surgicel use in the form of prolonged drainage following nephron-sparing surgery highlighting the technical predisposing factor with the aim to prevent undue resulting morbidity in future.

A 1.2-year-old female baby with features of left hemihypertrophy associated with complex cystic mass in upper pole of left kidney underwent partial nephrectomy. During partial nephrectomy hemostasis was achieved by ligation of vessels and surgicel was applied in multiple layers after application of Glubran 2 tissue glue. In postoperative period, an increase in drainage in the form of yellowish thick fluid approximately 5-10 ml in 24 hours was observed from fourth postoperative day. The biochemical analysis of fluid in form of creatinine and sodium values done twice on the fifth and eighth postoperative day, corresponded to serum values, thus confirming absence of urine leak. The microbiological analysis showed absence of inflammatory pathology. Thus, presence of thick yellowish sterile fluid was attributed to biodegradation of thick layer of surgicel and the correlation of drain output with ultrasonic thickness of surgicel showed progressive decrease in drainage by the end of second week, which coincided with decrease in ultrasonic thickness of surgicel. Thus, the serum values of fluid and the correlation of ultrasonic thickness with drain output provided evidence that excessive use of surgicel may be a contributing factor for prolonged drainage. The patient recovered well and is asymptomatic on follow-up for the last three months.

Since the use of oxidized cellulose by Frantz, sugicel has been used successfully as the biodegradable hemostatic agent.[1,2] Although exact mechanism of action is unclear, it appears to promote coagulation physically by providing mesh for platelets to start adhesion and aggregation.[1,2] Its biodegradation starts within 24 hours and depending on amount used and tissue bed, within 1 week multinucleated giant cells appear resulting in complete absorption in 4—8 weeks.[1,3,4] With its use in excess in abdominal and neurosurgical procedures, complications including postoperative granuloma, pseudo abscess, paraplegia, prolonged drainage, obstructive uropathy, etc. have been reported, but such experience in pediatric urology has rarely been reported till date.[1–3] As experienced in present and reported cases, surgicel has been found to be associated with yellowish thick discharge either in form of prolonged drainage or pseudo abscess like presentation.[1–3] Hence, although surgicel is a nonirritant biodegradable material but still because of its foreign nature it should be used in smallest amount as far as possible.[1] Thus, we recommend that during partial nephrectomy meticulous surgical technique aimed at securing visible bleeding vessels on renal surface along with application of thin or single layer of surgicel can avoid such unusual complication in future. The present experience thus can prove to be useful especially for younger practicing urologists performing major surgical procedures where panic attempts to control bleeding with improper use of surgicel can result in undue morbidity.

## References

[1] Mohamed FI, Christopher AP, Christopher PY (2002). A foreign body reaction to Surgicel mimicking an abscess following cardiac surgery. Eur J Cardiothorac Surg.

[2] Patanè F, Zingarelli E, Verzini A, Summa M (2001). Complication due to excessive use of Surgicel. Eur J Cardiothorac Surg.

[3] Sandhu GS, Elexpuru-Camiruaga JA, Buckley S (1996). Oxidized cellulose (Surgicel) granulomata mimicking tumour recurrence. Br J Neurosurg.

[4] Pierce AM, Wiebkin OW, Wilson DF (1984). Surgicel: Its fate following implantation. J Oral Pathology.

